# Anxiety Impacts Consent Capacity to Treatment in Alzheimer's Disease

**DOI:** 10.3389/fpsyg.2021.685430

**Published:** 2021-06-14

**Authors:** Yuka Kato, Teruyuki Matsuoka, Yoko Eguchi, Kiyoko Iiboshi, Hiroyuki Koumi, Kaeko Nakamura, Kayoko Okabe, Shutaro Nakaaki, Toshiaki A. Furukawa, Masaru Mimura, Jin Narumoto

**Affiliations:** ^1^Department of Psychiatry, Graduate School of Medical Science, Kyoto Prefectural University of Medicine, Kyoto, Japan; ^2^Department of Neuropsychiatry, Keio University School of Medicine, Tokyo, Japan; ^3^Department of Clinical Psychology, Faculty of Human Relations, Shigakukan University, Kagoshima, Japan; ^4^Department of Clinical Psychology, Faculty of Social Welfare, Hanazono University, Kyoto, Japan; ^5^Department of Psychiatry and Cognitive-Behavioral Medicine, Nagoya City University Graduate School of Medical Sciences, Nagoya, Japan; ^6^Department of Health Promotion and Human Behavior, Kyoto University School of Public Health, Kyoto, Japan

**Keywords:** decision making, capacity to consent to treatment, Alzheimer's disease, cognitive function, MacArthur Competence Assessment Tool, behavioral and psychological symptoms of dementia

## Abstract

This study aimed to clarify how behavioral and psychological symptoms of dementia (BPSD) and cognitive function affect the decision-making capacity of persons with Alzheimer's disease (AD) in a real informed consent situation about anti-dementia drug prescriptions. The participants were 76 patients with AD. We used the MacArthur Competence Assessment Tool to assess the capacity for consent to treatment (MacCAT-T). We simultaneously used the Mini-Mental State Examination, Executive Interview, Executive Clock Drawing Task, Logical Memory I of the Wechsler Memory Scale-Revised (LM I), LM II, and Neuropsychiatric Inventory (NPI) to assess cognitive function and psychiatric symptoms. We calculated the correlations between the MacCAT-T scores and the demographic, neuropsychological, and psychiatric variables. Once the univariable correlations were determined, we performed simple linear regression analyses to examine if the regression equations were significant. In the final analyses, we incorporated significant variables into stepwise multiple linear regression analyses to determine the most significant predictors of mental capacity. Age (β = −0.34), anxiety (β = −0.27), and LM I (β = 0.26) were significant predictors of “understanding” (adjusted *R*^2^ = 0.29). LM II (β = 0.39), anxiety (β = −0.29), and education (β = 0.21) were significant predictors of “understanding of alternative treatments” (adjusted *R*^2^ = 0.30). Anxiety (β = −0.36) and age (β = −0.22) were significant predictors of “appreciation” (adjusted *R*^2^ = 0.18). Age (β = −0.31) and anxiety (β = −0.28) were significant predictors of explained variance in “reasoning” (adjusted *R*^2^ = 0.17). Patients with anxiety had lower scores on all five MacCAT-T subscales: “understanding,” without 3.8 [*SD* = 1.2] vs. with 2.6 [*SD* = 1.1]; “understanding of alternative treatments,” without 2.9 [*SD* = 2.2] vs. with 1.3 [*SD* = 1.8]; “appreciation,” without 2.9 [*SD* = 1.1] vs. with 1.9 [*SD* = 1.2]; “reasoning,” without 4.0 [*SD* = 2.0] vs. with 2.7 [*SD* = 1.7]; and “expressing a choice,” without 1.9 [*SD* = 0.4] vs. with 1.5 [*SD* = 0.6]. Considering the effects of BPSD, cognitive function, and age/education when assessing consent capacity in persons with AD is important. Reducing anxiety may contribute to improved capacity in persons with AD.

## Introduction

At present, as the number of people with dementia encountered in clinical practice rapidly increases, providing treatment to cognitively impaired older patients is becoming problematic. Japan is the most aged country worldwide (Cabinet Office Government of Japan, 2019), and the increasing prevalence of dementia among the Japanese population is gaining attention. The latest report estimates that Japan will have approximately 7.3 million (20.6%) people with dementia by 2025 (Ninomiya, 2015). There has therefore been increasing interest in assessing older patients' healthcare decision-making capacity and decision support in Japan in recent years. Such assessment of capacity is essential to find a delicate balance between ensuring autonomy for people who can make decisions independently and providing protection to those with impaired decision-making capacity (Palmer and Harmell, 2016).

Previous research has indicated that the capacity to consent to treatment is reduced in individuals with Alzheimer's disease (AD) as compared to healthy individuals (Moye et al., 2006; Lui et al., 2010, 2012). An individual's consent capacity can be influenced by various factors, including cognitive function, psychiatric symptoms, personal values, and decision-making experience. Identifying the factors that influence consent capacity is important for developing effective interventions to support the capacity to consent. In particular, several studies have shown the association between decision-making capacity and cognitive functioning (Marson et al., 1996; Moye et al., 2006; Lui et al., 2010, 2012; Tallberg et al., 2013; Stormoen et al., 2014; Mueller et al., 2017). According to a systematic review about the capacity to consent to treatment in individuals with AD (van Duinkerken et al., 2018), executive functioning and processing speed (Okonkwo et al., 2007), total score on the AD Assessment Scale-Cognitive, category fluency, working memory, and processing speed (Lui et al., 2010, 2012), and episodic and working memory, processing speed, and verbal knowledge (Tallberg et al., 2013; Stormoen et al., 2014) are related to an individual's capacity to consent to treatment. In sum, deterioration of language, memory, processing speed, and executive function may negatively impact individuals' capacity to consent to treatment.

In addition to cognitive impairment, behavioral and psychological symptoms of dementia (BPSD) are also important factors that affect the capacity to consent to treatment (Mitoku and Shimanouchi, 2014; Bertrand et al., 2017). Mitoku and Shimanouchi (2014) explored the association between BPSD and decision-making levels and highlighted that individuals with BPSD demonstrated lower decision-making capacity than those without BPSD. Bertrand et al. (2017) explored the relationship between behavioral disturbance and the capacity to consent to treatment in 71 participants with AD by comparing those participants with and without BPSD. Participants with symptoms of delusions and apathy exhibited impaired expression of choice compared to their counterparts. Participants with euphoria as a symptom had more difficulty when discussing the consequences of treatment alternatives compared with patients without this symptom (Bertrand et al., 2017). However, few studies have focused on the impact of BPSD on individuals' capacity to consent to treatment. No studies have investigated the factors that affect decision-making capacity from both BPSD and cognitive function.

Most previous studies used hypothetical vignettes to evaluate the decision-making capacity (Marson et al., 1996; Moye et al., 2006; Lui et al., 2010, 2012; Tallberg et al., 2013; Stormoen et al., 2014). This capacity is situation-specific and must be assessed accordingly. It may be difficult for people with dementia to immerse themselves in a hypothetical scenario owing to their impaired abstraction capabilities (Haberstroh et al., 2014). Haberstroh et al. (2014) also indicated that hypothetical situations may frighten or confuse patients with dementia as they might imagine themselves to be genuinely afflicted with the hypothetical disease described. Hence, it is necessary to evaluate capacity in a real informed consent situation.

Therefore, this study aimed to investigate the influence of both BPSD and cognitive function on the capacity to consent to the prescription of anti-dementia drugs in participants with AD in a real informed consent situation, using multiple regression analyses.

## Methods

### Participants

This study was conducted from October 2, 2013 to March 30, 2018. Participants with AD (*n* = 76) who were older than 65 years were recruited from outpatients at memory clinics from three hospitals in Japan: the Kyoto Prefectural University of Medicine, the North Medical Center Kyoto Prefectural University of Medicine, and the Ujioubaku Hospital. The sample size was calculated using G^*^ Power 3.1 (Faul et al., 2009) for multiple linear regression analyses involving 20 predictors to detect an effect size (f2) of 0.25, with the power (1–β error probability) as 0.8 and the significant level (α error probability) as 0.05. The required sample size was 101 participants. However, because it was difficult to recruit more participants during the study period, the data from 76 patients were analyzed. Alzheimer's disease was diagnosed based on the criteria set by the National Institute of Neurological and Communicative Disease and Stroke-Alzheimer's Disease and Related Disorders Association for probable AD (McKhann et al., 1984). All participants had been comprehensively assessed by geriatric psychiatrists, had received brain magnetic resonance imaging or computed tomography, physical, and neurological examinations, and had reported their medical histories. Single photon emission computed tomography was added if necessary, to confirm the diagnosis. Clinical data, including functional status, were confirmed by family members or caregivers. Patients who were to start treatment with anti-dementia drugs were the inclusion criterion. The exclusion criteria included (i) a medical history of psychiatric disorders, traumatic brain injury, and alcohol or other substance abuse; (ii) an intellectual disability; and (iii) patients with severe impairment of visual acuity, auditory sensor, and/or communication. The Ethics Committee of the Kyoto Prefectural University of Medicine approved this study (ERB-E-18-3). All the participants and their substitute decisions-makers were provided with a study overview by the geriatric psychiatrists. A psychologist then gave them a complete description of the study, and the written informed consent was obtained from both the participants and their substitute decisions-makers.

### Instruments

#### MacArthur Competence Assessment Tool (MacCAT-T)

Participants' capacity to consent to treatment was assessed using the MacCAT-T (Grisso et al., 1997; Grisso and Appelbaum, 1998; Table 1), which takes 15–20 min to administer. It has excellent content validity, inter-rater reliability, and good test–retest reliability (Kim et al., 2001, 2007). It consists of four subscales: “understanding,” “appreciation,” “reasoning,” and “expressing a choice.” “Understanding” evaluates the extent to which the participant understands the disclosed medical condition and its treatment, along with the benefits and risks of treatment. “Appreciation” evaluates whether the participant acknowledges that the disclosed information applies to them and whether they recognize the possible benefits of the treatment. “Reasoning” evaluates whether the participant identifies the consequences of the treatment, whether they compare alternatives, whether they anticipate any effect on their everyday life, and whether their final choice follows logically from their own explanation. “Expressing a choice” evaluates whether the participant can clearly express their treatment choice. Participant responses were rated as follows: 2 points for adequate, 1 point for partially sufficient, and 0 points for insufficient. Total scores range as follows: Understanding, 0–6; Appreciation, 0–4; Reasoning, 0–8; and Expressing a choice, 0–2. The method does not provide a total score or a cutoff for competence, and evaluators must integrate the results with relevant clinical information to reach a judgment (Grisso and Appelbaum, 1998; Appelbaum, 2007).

**Table 1:** MacCAT-T script items.

**Understanding** ** (0–6)**	**Understanding of** ** alternative treatments** ** (0–8)**	**Appreciation** ** (0–4)**	**Reasoning** ** (0–8)**	**Expressing a choice** ** (0–2)**
Disease (0–2) 1. Diagnosis 2. Brain atrophy 3. Memory impairment 4. Disability in living independently 5. Slow progress	Benefits and risks (0–8) 1. No side effect 2. No difficulty taking medicine 3. Slow progress 4. Disability in living independently	Disease (0–2)	Reasoning (0–2)	Expressing a choice (0–2)
Treatment (0–2) 1. Drug name 2. Dosage 3. Type of medicine 4. Effect		Treatment (0–2)	Comparison (0–2)	
Benefits and risks (0–2) - Donepezil, galantamine, and rivastigmine) 1. Reducing memory impairment progression 2. Maintaining independent life 3. Nausea/Diarrhea 4. Insomnia - Memantine 1. Reducing memory impairment progression 2. Maintaining independent life 3. Dizziness/Lightheadedness 4. Sleepiness			Generate consequences (0–2)	
			Logical consistency (0–2)	

*MacCAT-T, MacArthur Competence Tool for Treatment*.

We wrote standardized scripts for the study, which included information about the diagnosis of dementia and treatment with anti-dementia drugs, particularly outlining the main features, benefits, and risks of treatment, as well as the no-treatment option. The three cholinesterase inhibitors, donepezil, galantamine, and rivastigmine, and the uncompetitive N-methyl-D-aspartate receptor antagonist memantine were included in the study as anti-dementia drugs. To make decisions easier for the participants, we selected only some, more frequent and important benefits and risks and offered an “understanding of alternative treatments” that only evaluated the benefits and risks of the no-treatment option.

#### Cognitive Function

For a general measure of cognitive function, we used the Japanese version of the Mini-Mental State Examination (MMSE-J; Folstein et al., 1975; Sugishita, 2012). It consists of 30 points, with lower scores indicating impaired cognition. The Japanese version of the Executive Interview (J-EXIT25; Royall et al., 1992; Matsuoka et al., 2014) was administered to evaluate executive function. It has 25 items and includes tests for frontal lobe function, such as the aural Trail Making Test, verbal and design fluency, interference task, primitive reflex, Go/No-Go task, and Luria hand sequences. Each item is rated on a score of 0–2, and the total score ranges from 0 to 50. A higher J-EXIT25 score indicates greater impairment. The Japanese version of the Executive Clock Drawing Task (J-CLOX; Royall et al., 1998; Matsuoka et al., 2014) was also administered. The J-CLOX has two parts: J-CLOX1, an unprompted Clock Drawing Task, is considered to involve executive function; and J-CLOX2, a copying task, is considered to involve visuospatial abilities (Royall et al., 1998; Matsuoka et al., 2014). Both parts are scored on a 15-point scale, with a lower score reflecting greater impairment. The Logical Memory (LM) of the Wechsler Memory Scale-Revised (WMS-R; Wechsler, 1987) was also applied. Short-term verbal memory was assessed using the LM I subtest of the WMS-R, while delayed verbal recall was assessed using the LM II subtest.

#### Psychiatric Symptoms

The Neuropsychiatric Inventory (NPI-12) was administered as a caregiver-based clinical instrument that evaluates neuropsychiatric symptoms of dementia (Cummings et al., 1994). It evaluates the existence of delusions, hallucinations, agitation, dysphoria, anxiety, euphoria, apathy, disinhibition, irritability, aberrant motor behavior, sleep disturbance, and eating problems. The frequency score ranges from 0 to 4 points, and the severity score ranges from 0 to 3 points. Each NPI subscale score is obtained by multiplying the frequency and severity scores, with a maximum score of 12. The total score ranges from 0 to 144 points, with a higher score indicating greater severity of symptoms. The Geriatric Depression Scale 15 items was used to screen for depressive symptoms (Sheikh and Yesavage, 1986; Watanabe and Imagawa, 2013). Activities of daily living were evaluated using the Physical Self-Maintenance Scale (PSMS) and the Instrumental Activities of Daily Living scale (Lowton and Brody, 1969).

#### Dementia Severity

Clinical Dementia Rating (CDR) is an observation scale for assessing six domains: memory, orientation, judgment, community affairs, home and hobbies, and personal care. It has the following scoring scale: CDR 0, healthy; CDR 0.5, questionable dementia; CDR 1, mild dementia; CDR 2, moderate dementia; and CDR3, severe dementia (Hughes et al., 1982).

### Procedure

Participants' cognitive function was assessed by trained clinical psychologists. Clinical Dementia Rating assessments were performed independently from cognitive assessments and the MacCAT-T by trained geriatric psychiatrists. The psychiatrist diagnosed AD, provided information about the disorder, and explained the advantages and disadvantages of anti-dementia drugs to the patient and their family. Thereafter, clinical psychologists assessed all the participants using the MacCAT-T. Information on dementia and anti-dementia drugs was given orally in the implementation of the MacCAT-T. If any of the participants could not respond adequately, we repeated the explanation and encouraged them to understand as much as possible. The MacCAT-T and cognitive assessments were conducted in a single session, which took a total of 40–60 min. In some cases, a break was given if the participants were tired.

To standardize MacCAT-T implementation and evaluation criteria, it was initially administered to nine participants as a pilot study. The first two participants were interviewed by YK. They were then independently scored using the MacCAT-T by five psychologists (including YK and KO) based on the interview recordings. Then, a study session was conducted in which the implementation and evaluation criteria based on the scoring results was discussed until the standard was determined. The next seven participants were interviewed by one of the five psychologists (including YK and KO). Each participant was scored separately based on the recordings. We discussed the implementation and evaluation criteria again and prepared a scoring manual.

### Statistical Analysis

Data were analyzed using SPSS 26.0 J for Windows (IBM Corp., Armonk, NY, USA.). *P* < 0.05 was considered significant. To understand the clinical significance of the MacCAT-T scores, Spearman correlation coefficients between the MacCAT-T and the demographic variables, CDR, neuropsychological, and psychiatric variables were calculated. Once the univariable correlations were determined, we performed simple linear regression analyses to examine if the regression equations were significant. In the final analyses, we incorporated significant variables into a stepwise multiple linear regression analyses to determine the most significant predictors of mental capacity. In addition, for NPI items with significant univariate correlations, participants were divided into two groups based on the presence or absence of neuropsychiatric symptoms for each NPI subscale. Then, we compared the scores on each scale of the MacCAT-T scores between the two groups using the Mann–Whitney U-test.

## Results

### Demographic and Clinical Characteristics of Participants

Seventy-six participants (22 men and 54 women) participated in the study. The mean education history of the participants was 10.6 (Standard Deviation; *SD* = 2.9) years. The classification of anti-dementia drugs was 55 donepezil (72.37%), 11 galantamine (14.47%), 5 rivastigmine (6.58%), and 5 memantine (6.58%). Demographic and neuropsychological data for each CDR group are shown in Table 2. Forty-nine of these (64.47%) were considered as CDR 1.

**Table 2:** Demographic and clinical characteristics.

	**All**	**CDR 0.5**	**CDR1**	**CDR 2**
	**(*n* = 76)**	**(*n* = 19)**	**(*n* = 49)**	**(*n* = 8)**
	**Mean (*SD*)**	**Mean (*SD*)**	**Mean (*SD*)**	**Mean (*SD*)**
Age (years)	79.7 (6.2)	78.7 (5.5)	79.6 (6.2)	82.9 (7.6)
Sex (male/female)	22/54	5/14	15/34	2/6
Education (years)	10.6 (2.9)	11.2 (2.9)	10.4 (2.9)	10.6 (3.5)
MMSE-J (0–30)	21.0 (3.4)	23.3 (3.4)	20.7 (3.0)	17.8 (2.0)
J-EXIT25 (0–50)	16.8 (5.9)	14.2 (5.6)	17.4 (5.7)	19.4 (6.3)
J-CLOX1 (0–15)	9.1 (3.6)	9.6 (3.7)	9.2 (3.4)	7.6 (4.3)
J-CLOX2 (0–15)	13.0 (2.4)	12.9 (2.8)	13.3 (1.9)	11.4 (3.9)
LM I (0–50)	3.6 (3.4)	5.4 (3.8)	3.2 (3.2)	1.9 (1.6)
LM II (0–50)	0.8 (1.7)	0.9 (1.9)	0.8 (1.7)	0.3 (0.5)
PSMS (0–6)	5.4 (1.0)	5.8 (0.5)	5.6 (0.8)	3.8 (1.5)
IADL (male; *n* = 22) (0–3)	2.3 (1.2)	2.0 (0.7)	2.3 (1.3)	2.5 (2.1)
IADL (female; *n* = 54) (0–8)	5.9 (1.8)	6.5 (1.5)	6.1 (1.5)	3.0 (2.0)
GDS (0–15)	4.5 (3.1)	5.2 (3.3)	4.3 (3.2)	3.9 (2.5)
NPI-12 (0–144)	7.4 (11.0)	5.1 (6.9)	6.7 (10.2)	17.3 (18.2)

*CDR, Clinical Dementia Rating; GDS, Geriatric Depression Scale; IADL, Instrumental Activities of Daily Living; J-EXIT25, Japanese version of Executive Interview; J-CLOX, Japanese version of Executive Clock Drawing Task; LM, Logical Memory, MMSE-J, Japanese version of Mini-Mental State Examination; NPI, Neuropsychiatric Inventory; PSMS, Physical Self-Maintenance Scale; SD, standard deviation*.

### MacCAT-T Performance

MacArthur Competence Tool for Treatment performances for all participants and for each CDR group are shown in Table 3. For all participants with AD as a whole, the mean “expressing a choice” was 1.8 (*SD* = 0.5), which is almost perfect, indicating that most people with AD could state a clear treatment choice. In contrast, “understanding: benefits and risks (mean = 0.7; *SD* = 0.5),” “understanding of alternative treatments (mean = 2.6; *SD* = 2.2),” “reasoning: comparison (mean = 0.7; *SD* = 0.7),” and “reasoning: generate consequences (mean = 0.9; *SD* = 0.7)” did not meet half of the full score determined for each item.

**Table 3:** MacCAT-T performance.

	**All**	**CDR 0.5**	**CDR1**	**CDR 2**
	**(*n* = 76)**	**(*n* = 19)**	**(*n* = 49)**	**(*n* = 8)**
	**Mean (*SD*)**	**Mean (*SD*)**	**Mean (*SD*)**	**Mean (*SD*)**
**Understanding** (0–6)	3.5 (1.2)	4.0 (1.1)	3.5 (1.2)	2.4 (1.5)
Disease (0–2)	1.3 (0.5)	1.4 (0.5)	1.3 (0.5)	1.0 (0.4)
Treatment (0–2)	1.5 (0.5)	1.7 (0.3)	1.5 (0.4)	1.1 (0.7)
Benefits and risks (0–2)	0.7 (0.5)	0.9 (0.5)	0.7 (0.5)	0.3 (0.5)
**Understanding of alternative treatments** (0–8)	2.6 (2.2)	3.6 (2.3)	2.3 (2.0)	1.9 (2.3)
**Appreciation** (0–4)	2.7 (1.2)	2.6 (1.1)	2.9 (1.2)	1.9 (1.6)
Disease (0–2)	1.4 (0.7)	1.4 (0.8)	1.4 (0.7)	1.0 (0.9)
Treatment (0–2)	1.3 (0.7)	1.3 (0.7)	1.5 (0.6)	0.9 (0.8)
**Reasoning** (0–8)	3.7 (2.0)	4.0 (2.0)	3.7 (1.9)	3.4 (2.3)
Reasoning (0–2)	1.0 (0.6)	1.0 (0.5)	0.9 (0.6)	1.1 (0.6)
Comparison (0–2)	0.7 (0.7)	0.8 (0.8)	0.6 (0.7)	0.6 (0.7)
Generate consequences (0–2)	0.9 (0.7)	0.9 (0.8)	0.9 (0.7)	0.6 (0.7)
Logical consistency (0–2)	1.2 (0.7)	1.3 (0.7)	1.2 (0.6)	1.0 (0.8)
**Expressing a choice** (0–2)	1.8 (0.5)	1.7 (0.6)	1.8 (0.5)	1.9 (0.4)

*SD, standard deviation*.

### Associations With Demographic, Neuropsychological, and Psychiatric Variables

Correlations between MacCAT-T scores and demographic and cognitive variables and NPI symptoms are presented in Table 4. “Understanding” was significantly correlated with age, education, CDR, MMSE-J, J-EXIT25, J-CLOX1, LM I, LM II, and anxiety. “Understanding of alternative treatments” was significantly correlated with age, education, CDR, MMSE-J, J-CLOX1, LM I, and LM II, and anxiety. “Appreciation” was significantly correlated with age, education, and anxiety. “Reasoning” was significantly correlated with age, education, and anxiety. “Expressing a choice” was significantly correlated only with anxiety.

**Table 4:** Spearman correlations between the MacCAT-T and demographic and cognitive variables and NPI symptoms.

		**Understanding**	**Understanding of** ** alternative treatments**	**Appreciation**	**Reasoning**	**Expressing a choice**
Demographic variables	Age	−0.33 (−0.51 to −0.11)^*^	−0.28 (−0.48 to −0.06)^*^	−0.25 (−0.45 to −0.03)^*^	−0.30 (−0.49 to −0.08)^*^	−0.05 (−0.27 to 0.18)
	Education	0.30 (0.08 to 0.49)^*^	0.27 (0.05 to 0.47)^*^	0.23 (0.00 to 0.43)^*^	0.25 (0.03 to 0.45)^*^	0.18 (−0.05 to 0.39)
CDR		−0.31 (−0.50 to −0.10^*^)	−0.27 (−0.46 to −0.04)^*^	−0.04 (−0.26 to 0.19)	−0.09 (−0.31 to 0.13)	0.05 (−0.18 to 0.27)
Cognitive variables	MMSE-J	0.38 (0.16 to 0.55)^*^	0.23 (0.00 to 0.43)^*^	0.07 (−0.16 to 0.29)	0.10 (−0.13 to 0.32)	0.15 (−0.08 to 0.36)
	J-EXIT25	−0.31 (−0.50 to −0.09)^*^	−0.20 (−0.41 to 0.03)	−0.02 (−0.25 to 0.21)	−0.15 (−0.37 to 0.08)	−0.15 (−0.36 to 0.08)
	J-CLOX1	0.33 (0.11 to 0.52)^*^	0.23 (0.00 to 0.43)^*^	0.13 (−0.10 to 0.35)	0.10 (−0.13 to 0.32)	0.07 (−0.16 to 0.29)
	J-CLOX2	0.06 (−0.17 to 0.28)	0.18 (−0.05 to 0.39)	−0.01 (−0.23 to 0.22)	−0.00 (−0.23 to 0.22)	0.12 (−0.11 to 0.34)
	LM I	0.37 (0.16 to 0.55)^*^	0.33 (0.12 to 0.52)^*^	−0.04 (−0.26 to 0.19)	0.18 (−0.05 to 0.39)	0.04 (−0.19 to 0.26)
	LM II	0.32 (0.11 to 0.51)^*^	0.41 (0.20 to 0.58)^*^	0.21 (−0.01 to 0.42)	0.18 (−0.05 to 0.39)	0.04 (−0.19 to 0.26)
NPI-12	Delusions	0.07 (−0.16 to 0.29)	−0.01 (−0.23 to 0.22)	−0.04 (−0.27 to 0.18)	0.17 (−0.06 to 0.38)	−0.15 (−0.36 to 0.08)
	Hallucinations	−0.22 (−0.42 to 0.01)	−0.13 (−0.35 to 0.10)	−0.18 (−0.39 to 0.05)	−0.07 (−0.29 to 0.16)	−0.12 (−0.34 to 0.11)
	Agitation	0.16 (−0.06 to 0.38)	0.01 (−0.22 to 0.23)	0.05 (−0.18 to 0.27)	0.01 (−0.21 to 0.24)	0.09 (−0.14 to 0.31)
	Dysphoria	0.12 (−0.11 to 0.34)	0.05 (−0.18 to 0.27)	0.17 (−0.06 to 0.38)	0.17 (−0.06 to 0.38)	−0.07 (−0.29 to 0.16)
	Anxiety	−0.40 (−0.58 to −0.19)^*^	−0.35 (−0.53 to −0.14)^*^	−0.37 (−0.55 to −0.16)^*^	−0.28 (−0.47 to −0.06)^*^	−0.29 (−0.48 to −0.06)^*^
	Euphoria	−0.12 (−0.34 to 0.11)	−0.01 (−0.23 to 0.22)	−0.09 (−0.31 to 0.14)	0.04 (−0.19 to 0.26)	0.13 (−0.10 to 0.35)
	Apathy	−0.11 (−0.32 to 0.12)	−0.06 (−0.28 to 0.17)	0.00 (−0.23 to 0.22)	−0.11 (−0.33 to 0.12)	−0.02 (−0.25 to 0.20)
	Disinhibition	−0.03 (−0.26 to 0.19)	0.05 (−0.18 to 0.27)	−0.12 (−0.33 to 0.11)	0.06 (−0.17 to 0.28)	0.12 (−0.11 to 0.33)
	Irritability	0.01 (−0.21 to 0.24)	−0.01 (−0.23 to 0.22)	0.07 (−0.16 to 0.29)	0.03 (−0.20 to 0.25)	−0.06 (−0.28 to 0.17)
	Aberrant motor behavior	0.03 (−0.20 to 0.25)	0.12 (−0.11 to 0.33)	−0.11 (−0.33 to 0.12)	0.14 (−0.09 to 0.35)	0.10 (−0.13 to 0.32)
	Sleep disturbance	−0.10 (−0.32 to 0.13)	−0.02 (−0.25 to 0.20)	−0.03 (−0.25 to 0.20)	−0.06 (−0.28 to 0.17)	0.13 (−0.10 to 0.35)
	Eating problems	0.14 (−0.08 to 0.36)	−0.03 (−0.26 to 0.19)	0.05 (−0.18 to 0.27)	−0.03 (−0.26 to 0.19)	0.03 (−0.2 to 0.25)

*95% confidence intervals in brackets. CDR, Clinical Dementia Rating; J-EXIT25, Japanese version of Executive Interview; J-CLOX, Japanese version of Executive Clock Drawing Task; LM, Logical Memory; MMSE-J, Japanese version of Mini-Mental State Examination; MacCAT-T, MacArthur Competence Tool for Treatment; NPI, Neuropsychiatric Inventory*.

* *significant*.

Simple linear regression analyses indicated that age, education, CDR, MMSE-J, J-EXIT25, J-CLOX1, LM I, and anxiety contributed significantly to “understanding.” Age, education, MMSE-J, J-CLOX1, LM I, and LM II, and anxiety contributed significantly to the “understanding of alternative treatments.” Age and anxiety contributed significantly to “Appreciation” and “Reasoning.” Multiple linear regression analyses using these variables indicated that age, anxiety, and LM I explained the variance in “understanding.” LM II, anxiety, and education explained the variance in “understanding of alternative treatments.” Anxiety and age explained the variance in “appreciation.” Age and anxiety explained the variance in “reasoning.” “Expressing a choice” was excluded from the regression analysis. These results are shown in Table 5.

**Table 5:** Simple linear regression and stepwise linear regression analyses with significant demographic, neuropsychological, and psychiatric variables for decision-making abilities.

**Dependent variables**	**Independent** ** variables**	**Simple linear regression analyses**			**Stepwise linear regression analyses**				
		**β**	**95% CI**	***P***	***β***	**95% CI**	***P***	***VIF***	**Adjusted *R*^**2**^**
Understanding	Age	−0.39	−0.12 to −0.04	<0.001	−0.34	−0.11 to −0.03	0.001	1.02	
	Anxiety	−0.36	−0.38 to −0.09	0.002	−0.27	−0.31 to −0.04	0.009	1.05	0.29
	LM I	0.34	0.05 to 0.21	0.002	0.26	0.02 to −0.17	0.011	1.04	
	Education	0.29	0.03 to 0.22	0.011	—	—	—	—	—
	CDR	−0.35	−1.71 to −0.39	0.002	—	—	—	—	—
	MMSE-J	0.38	0.06 to 0.22	0.001	—	—	—	—	—
	J-EXIT25	−0.28	−0.11 to −0.01	0.014	—	—	—	—	—
	J-CLOX1	0.34	0.04 to 0.20	0.002	—	—	—	—	—
	LM II	0.22	−0.00 to 0.33	0.051	—	—	—	—	—
Understanding of alternative treatments	LM II	0.43	0.29 to 0.83	<0.001	0.39	0.25 to 0.76	<0.001	1.02	
	Anxiety	−0.35	−0.65 to −0.16	0.002	−0.29	−0.56 to −0.11	0.004	1.02	0.30
	Education	0.25	0.02 to 0.36	0.028	0.21	0.01 to 0.30	0.036	1.01	
	Age	−0.28	−0.18 to −0.02	0.016	—	—	—	—	—
	CDR	−0.23	−2.39 to 0.01	0.051	—	—	—	—	—
	MMSE-J	0.25	0.02 to 0.31	0.030	—	—	—	—	—
	J-CLOX1	0.25	0.02 to 0.29	0.027	—	—	—	—	—
	LM I	0.33	0.07 to 0.35	0.004	—	—	—	—	—
Appreciation	Anxiety	−0.39	−0.38 to −0.11	0.001	−0.36	−0.37 to −0.10	0.001	1.01	
	Age	−0.27	−0.10 to −0.01	0.021	−0.22	−0.09 to −0.00	0.037	1.01	0.18
	Education	0.20	−0.01 to 0.18	0.087	—	—	—	—	—
Reasoning	Age	−0.34	−0.18 to −0.04	0.003	−0.31	−0.17 to −0.03	0.005	1.01	
	Anxiety	−0.32	−0.56 to −0.10	0.005	−0.28	−0.51 to −0.07	0.009	1.01	0.17
	Education	0.23	0.00 to 0.31	0.047	—	—	—	—	—
Expressing a choice	Anxiety	−0.13	−0.09 to 0.03	0.267	—	—	—	—	—

*CDR, Clinical Dementia Rating; CI, Confidence Interval; J-EXIT25, Japanese version of Executive Interview; J-CLOX, Japanese version of Executive Clock Drawing Task; LM, Logical Memory; MMSE-J, Japanese version of Mini-Mental State Examination; VIF, Variance Inflation Factor*.

**Figure 1:**
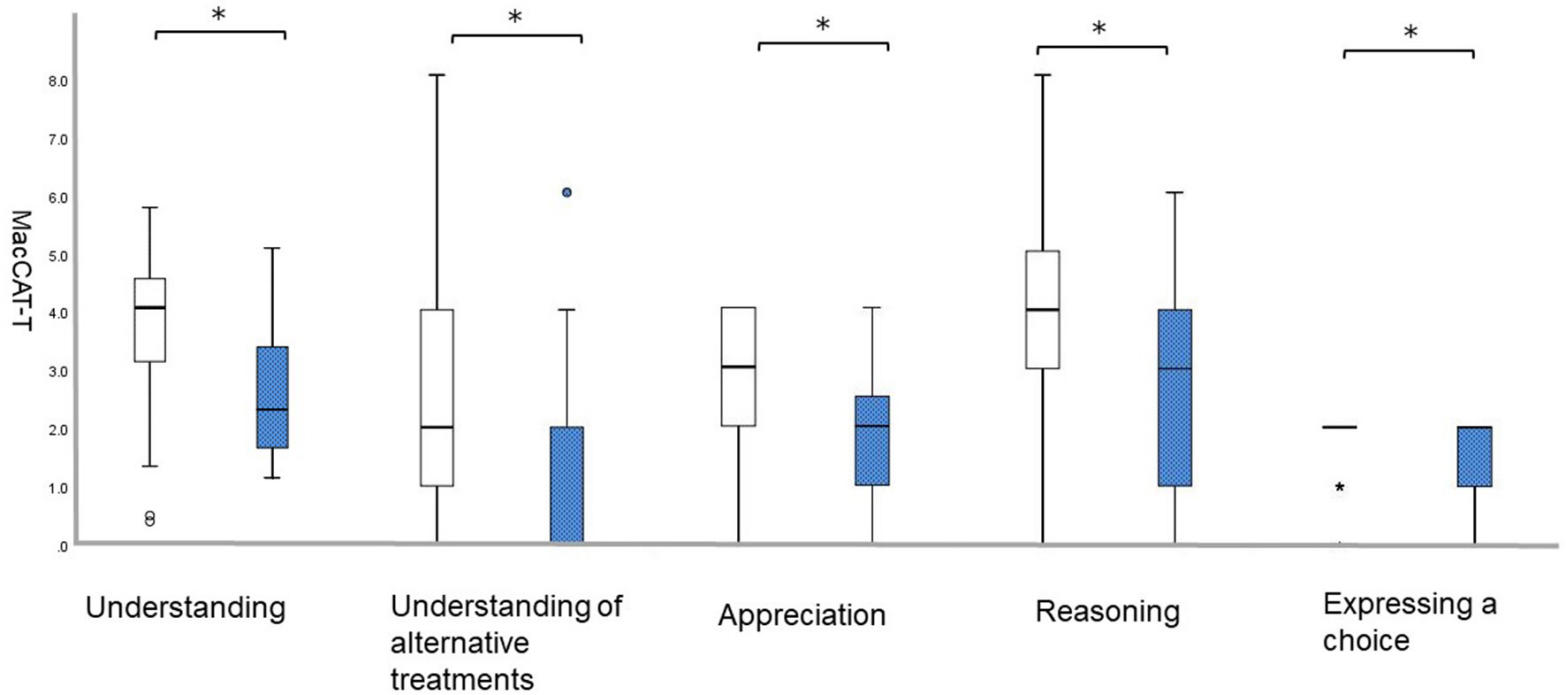
Differences on the MacCAT-T subscales according to NPI-12. White bars represent patients with a score of 0 on anxiety; blue bars represent those with a score of 1 or higher on anxiety.; *p < 0.05. MacCAT-T, MacArthur Competence Tool for Treatment; NPI, Neuropsychiatric Inventory.

The frequency of individuals without/with anxiety was 60/16 (78.94/21.05%). Individuals with anxiety had lower scores on all five MacCAT-T subscales: “understanding,” without 3.8 [*SD* = 1.2] vs. with 2.6 [*SD* = 1.1], *U* = 216.0, *p* = 0.001; “understanding of alternative treatments,” without 2.9 [*SD* = 2.2] vs. with 1.3 [*SD* = 1.8], *U* = 261.5, *p* = 0.005; “appreciation,” without 2.9 [*SD* = 1.1] vs. with 1.9 [*SD* = 1.2], *U* = 248.0, *p* = 0.002; “reasoning,” without 4.0 [*SD* = 2.0] vs. with 2.7 [*SD* = 1.7], *U* = 306.5, *p* = 0.025; and “expressing a choice,” without 1.9 [*SD* = 0.4] vs. with 1.5 [*SD* = 0.6], *U* = 333.5, *p* = 0.007 (Figure 1).

## Discussion

### Characteristics of Capacity to Consent of Participants With AD

Our results revealed that “understanding of alternative treatments,” “understanding: benefits and risks,” “reasoning: comparison,” and “reasoning: generate consequences” scores were less than half of that required for each item. Thus, these items were particularly difficult for individuals with AD. The result that participants with dementia have impaired “understanding” and “reasoning” is consistent with a previous study (Mueller et al., 2017). In particular, “understanding” is considered as a fundamental element in ensuring patients' capacity to consent to treatment (Owen et al., 2013), and it is therefore important to carefully confirm patients' degree of understanding when providing support to enhance their understanding during the decision-making process. On the other hand, most participants in the study could express a treatment choice, which is considered a strength in the context of the study. This finding is also consistent with previous studies (Gurrera et al., 2006; Moye et al., 2006; Lui et al., 2012; Mueller et al., 2017). However, participants' expression of treatment choice is not always supported by sufficient understanding, appreciation, and reasoning. These findings show that a distinctly communicated treatment option does not in itself convey capacity to consent (Moye et al., 2006). Therefore, we must ensure that patients explain what they have understood in their own words.

### Impact of BPSD and Cognitive Function on the Capacity to Consent

As a new finding, our study showed that anxiety was negatively correlated with all five MacCAT-T subscale scores as individuals with anxiety had lower scores on all subscales. Multiple regression analyses showed that age, anxiety, and LM I were significant predictors of “understanding,” and LM II, anxiety, and education were significant predictors of “understanding of alternative treatments.” On the other hand, only anxiety and age were significant predictors of “appreciation” and “reasoning.” This study emphasized the effect of anxiety on the capacity of individuals with AD to consent to treatment.

Anxiety may lead to reduced attention and concentration and deterioration of understanding. Clinical and animal behavioral studies have long established that increased anxiety can cause cognitive impairments, including an effect on decision-making ability or impaired behavioral flexibility (Park and Moghaddam, 2017). Previous studies have noted that anxiety leads to biased attention to threat-related stimuli and results in heightened distractibility owing to non-threatening stimuli, as suggested by reduced concentration and multi-tasking capability in anxious individuals (Eysenck et al., 2007). Additionally, Hartley and Phelps (2012) reported that anxiety increases attention to negative choice options, along with the likelihood of ambiguous options being interpreted negatively and the tendency to avoid potential negative outcomes, even at the cost of losing potential gains. Patients with high anxiety may therefore overestimate the disadvantages of treatment rather than its benefits, or easily refuse treatment altogether to avoid its fear and side effects. These findings thus suggest that reducing anxiety may contribute to the improvement of capacity to consent in persons with AD. Furthermore, anxiety was evaluated by the NPI-12—a caregiver-based instrument; however, in clinical settings, it may be useful to perform a self-administered anxiety scale, not just for the caregiver, to assess the level and content of the patient's anxiety when conducting evaluation of the consent to treatment. This approach is because it is possible to consider their condition more carefully. In a systematic review about the ways to evaluate anxiety symptoms in persons with dementia (Goodarzi et al., 2019), three validated tools were identified: the Geriatric Anxiety Inventory, Penn State Worry Questionnaire, and the Rating Anxiety in Dementia (RAID) scale. Goodarzi et al. (2019) reported that the RAID has the highest sensitivity for anxiety and was specifically designed for those experiencing dementia; thus, it may be useful to take advantage of these scales. However, information about the impact of anxiety on capacity to consent to treatment in individuals with dementia is insufficient. Bertrand et al. (2017) suggested that individuals with AD who show symptoms of delusions and apathy exhibit impaired expression of choice in comparison to those without these symptoms. Furthermore, individuals with AD who indicate euphoria as a symptom had more difficulties discussing the consequences of treatment alternatives compared to those without this symptom (Bertrand et al., 2017). However, no between-group differences in capacity were observed for patients with anxiety in the study (Bertrand et al., 2017). (Larkin and Hutton, 2017), who conducted a systematic review about the capacity to consent to treatment in patients with psychosis, reported that state and trait anxiety may be positively associated with aspects of capacity to consent to treatment; thus, greater anxiety was associated with greater capacity to consent. However, individuals with dementia were not included in this review. As few studies have investigated the association between BPSD and capacity to consent to treatment in persons with AD, further research is needed. As Bertrand et al. (2017) noted, a longitudinal study may be useful to deepen our understanding of this association.

Regarding the effects of cognitive function, LM I was a significant predictor in “understanding” and LM II was a significant predictor in the “understanding of alternative treatments.” Previous studies that investigated the association between cognitive function and the consent capacity reported that the element of understanding was most associated with cognitive function (Gurrera et al., 2006; Mueller et al., 2017). It is interesting that this study, which examined the influence of both BPSD and cognitive function, also showed that cognitive function was involved. For “understanding,” it is important that patients remember the content immediately after hearing the explanation. On the contrary, the “understanding of alternative treatments” appeared to be more stressful on delayed memory than “understanding.” Even in a clinical setting, it was more difficult for some participants to understand the benefits and risks of the no-treatment option than the explanation for the treatment option because they were confused between both explanations. Understanding of the alternative treatments could be improved by reducing the burden of delayed memory through the use of memory aids.

Age was a significant predictor in “understanding,” “appreciation,” and “reasoning,” and education was a significant predictor in the “understanding of alternative treatments”—similar to or even higher than anxiety. These results indicate that the influence of age and education should be considered in addition to BPSD and cognitive function in the evaluation of consent capacity. Lui et al. (2012) reported the effect of age and education on the capacity to provide consent. Thus, we should pay more attention to the level of consent capacity of the people with dementia as they get older or if they are less educated.

### Support the Capacity to Consent

Finally, we present some examples that support the capacity to consent to treatment of people with AD. We need to strive to optimize the capacity to consent to treatment of patients with dementia by providing specific cognitive and psychological supports that are related to impaired capacity.

Rubright et al. (2010) demonstrated that a one-page memory and organization aid led to improved research consent capacity: the understanding of the MacCAT for Clinical Research (MacCAT-CR) of persons with very mild to moderate AD. As mentioned above, in our study, “understanding” was one of the difficult tasks for participants with AD. Strategies, such as the aid, that simplify the presentation and discussion of information may lead to improved capacity to consent to treatment for individuals with AD because it can reduce demands on participants' memory and attention during the task. The use of visual tools such as pictures, diagrams, and photographs, along with information provided using bullet points may also be useful. Recently, the development of a decision-aid has been attracting attention. Murphy and Oliver (2013) reported that the use of Talking Mats, which uses a simple system of picture symbols as a low-technology communication framework, was useful for people with dementia when making decisions about their daily lives. Mueller et al. (2019) also reported that decision support, in terms of providing explicit information about the risk involved in initially preferred options, can improve decision-making under objective risk conditions in persons with mild AD. This finding is particularly interesting because it can be applied to medical consent situations such as choosing between medications with different levels of side effect risks. Consequently, developing decision aids is desirable to help people with dementia make health decisions.

Considering the psychological aspects of people with dementia who are facing decision-making, taking enough time to relieve their anxiety and having someone trustworthy attending to the decision-making situation is equally important. It is also necessary to consider delaying making the decision until BPSD is more manageable. According to Hamann et al. (2011), persons with AD prefer to participate in healthcare-related decisions, particularly in social ones. It is, therefore, necessary to accumulate findings that improve their capacity to consent to treatment through further research.

## Study Limitations

This study had several limitations. First, the number of participants was less than the recommended sample size provided by G^*^Power. Second, normal control data were unavailable because healthy individuals were not included. This aspect is the limitation associated with real decision-making. Third, we did not collect data on the duration of AD and medical follow-up. Fourth, we cannot determine the causal relationship between decision-making capacity and BPSD and cognitive function because our design was cross-sectional. Finally, we did not distinguish between general anxiety and illness-specific anxiety.

## Conclusion

Our study showed that age, anxiety, and LM I were significant predictors of “understanding;” and LM II, anxiety, and education were significant predictors of the “understanding of alternative treatments.” On the other hand, only anxiety and age were significant predictors of “appreciation” and “reasoning.” Therefore, we suggest that considering the effects of BPSD, cognitive function, and age/education when assessing consent capacity in persons with AD is important. This study emphasized the effect of anxiety on the capacity of persons with AD to consent to treatment. Reducing anxiety may improve the capacity. Identifying factors that affect the capacity may enable the development of clinically useful theoretical models, which, in turn, may help develop effective interventions in this regard.

## Data Availability Statement

The raw data supporting the conclusions of this article will be made available by the authors, without undue reservation. Requests to access the datasets should be directed to Yuka Kato, y-kato@koto.kpu-m.ac.jp.

## Ethics Statement

The studies involving human participants were reviewed and approved by the Ethics Committee of the Kyoto Prefectural University of Medicine (ERB-E-18-3). The patients/participants provided their written informed consent to participate in this study.

## Author Contributions

YK designed the study, collected and analyzed the data, and wrote the paper. JN designed the study, collected the data, and wrote the paper. TM designed the study, collected the data, and assisted with the writing of the paper. KN and KO collected the data and assisted with the writing of the paper. YE, KI, HK, SN, and MM designed the study and assisted with the writing of the paper. TAF supervised the statistical design of the study and assisted with the writing of the paper. All authors revised and approved the final manuscript and are accountable for all aspects of the work.

## Conflict of Interest

The authors declare that the research was conducted in the absence of any commercial or financial relationships that could be construed as a potential conflict of interest.
